# Fingerprint Spectrophotometric Methods for the Determination of Co-Formulated Otic Solution of Ciprofloxacin and Fluocinolone Acetonide in Their Challengeable Ratio

**DOI:** 10.1155/2019/8919345

**Published:** 2019-09-08

**Authors:** Reem H. Obaydo, Amir Alhaj Sakur

**Affiliations:** Analytical and Food Chemistry Department, Faculty of Pharmacy, Aleppo University, Aleppo, Syria

## Abstract

Six spectrophotometric methods were developed to determine a new single-dose otic solution known as “Otovel®,” which consists of two components: the major one is ciprofloxacin (CIP) and the minor is fluocinolone acetonide (FLU). The ratio of (CIP) and (FLU) in Otovel® is 12 : 1, which is considered a challengeable ratio for UV determination. Thus, spectrum addition as a sample enrichment technique was required for the analysis of (FLU) low concentration. All these methods were capable of resolving the spectra for each component in *D* ^0^ belonging to the fingerprint resolution technique. The former absorptivity centering (a-centering) method was recently developed in 2018; it was effectively applied for its solution of both binary components in Otovel®, while another method, ratio subtraction (RS), is considered as an original resolution method that could be applied to determine only one component in mixtures. However, the other four methods that are related to their original method (RS) were extended ratio subtraction (EXRS), constant multiplication (CM), unified constant subtraction (UCS), and spectrum subtraction (SS). They were also easily applied for completing the quantification of binary mixture drugs present in Otovel®. The linearity ranges were found to be 3.0–15.0 *μ*g/mL for (CIP) and (FLU), respectively. All results acquired from the proposed methods were successfully estimated according to ICH criteria and were statistically compared with official ones where no differences were noticed.

## 1. Introduction

Recently many pharmaceutical companies have provided a fixed-dose combination instead of monotherapy for the treatment of certain diseases, offering more competence and less cost and decreasing the side effects associated with the treatment.

Otovel® is considered an example of fixed-dose antibiotic-corticosteroid combination otic drops, which has been recently approved by the FDA for otorrhea treatment [1]. Various studies have reported that the topical antibiotic-corticosteroid drops are more effective in therapy than both antibiotic alone and glucocorticoid treatment on its own [2, 3].

Ciprofloxacin and fluocinolone acetonide have been recently co-formulated in Otovel® otic solution; the ratio of the two proposed drugs is 12 : 1, respectively. This ratio limits the analysis of this preparation; thus, spectrum addition technique [4] was applied to increase and determine the amount of fluocinolone acetonide.

**Figure 1:**
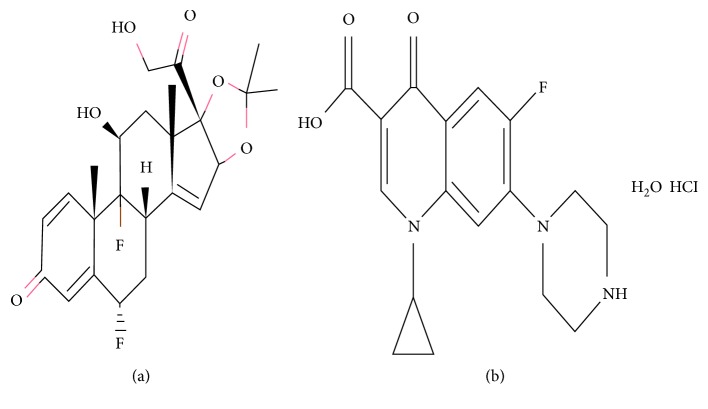
Chemical structure of fluocinolone acetonide (a) and ciprofloxacin hydrochloride monohydrate (b).

Ciprofloxacin hydrochloride (CIP) belongs to a class of drugs called quinolone antibiotics that work by stopping the growth of bacteria. Fluocinolone acetonide (FLU) is a medium-strength corticosteroid, which has anti-inflammatory, antipruritic, and vasoconstrictive properties [5–7]. The structures are shown in Figure 1.

Literature survey revealed that (CIP) has been determined as a single component using spectrophotometry [8–10], thin layer chromatography [11, 12], HPLC [13, 14], capillary electrophoresis [15, 16], and chemometric method [17, 18]. Estimation of (FLU) as single formulation was accomplished using spectrophotometry [19], gas chromatography [20], thin layer chromatography [21], and HPLC [22–27].

Recently, the literature reports only one study for the concurrent determination of (CIP) and (FLU) in Otovel® by the same authors of the present work. In the study [28], spectrophotometric methods depending on presence of isosbestic points have been implemented for the simultaneous determination of both drugs in Otovel®. However, dealing with isosbestic-point spectrophotometric methods requires two regression equations at least to complete the analysis. But, these isosbestic-point spectrophotometric techniques could not achieve the resolution of the spectra with multicomponent mixtures in zero-order form, and as a result, this technique could not be used to get the resolved spectral profile for each component in mixtures.

Spectrophotometric methods based on isosbestic point (in zero order, ratio, or derivative) for resolving binary mixtures with minor components required derivative steps, calculating the area under the curve, and obtaining ratio spectra, and this makes these methods need multiple steps to accomplish the analysis of the co-formulated drugs.

The reported methods in [28] are based on leveling effect of the isoabsorptive point in binary mixture with minor component. All methods require presence of isoabsorptive point in spectra as well as the concentration of the minor component calculated based on the difference between the total concentration and the concentration of the major one, and thus, making any mistake in the determination of the total concentration and the major one reflects on the determination of the minor component. So, the reported work fails to give a special regression equation for determination the minor component and its concentration was calculated by difference between the total concentration and the major one.

The determination and analysis of co-formulated drug mixtures is a daily challenge for drug analysts. Academic researchers in the analytical field must deal with new proposals to solve several problems faced by analysts.

Through this investigation, the fingerprint resolution techniques were suggested to get more accurate results for the determination of minor component (FLU) presenting in the binary mixture of Otovel®. In addition, the novel factorized spectrum was also suggested to be used as an innovative resolution tool for analysis of mixtures.

These methods were based on utilizing the absorptivity factor or constants of the ratio spectra which eliminate the spectral overlapping without prior separation and do not require search for zero-crossing points.

In terms of quality control and for all quantitative analysis, peak purity is a major task, which can be adopted in different ways; the fingerprint resolution techniques are one of the most important approaches which is used in quality control laboratories. An essential requisite of these techniques is the ability to get the recovered zero-order absorption spectrum of each drug separately from the mixture of the drugs with overlapped spectra, and as a result, the purity of the separated spectra could be verified. The advantage of using the fingerprint resolution technique is the ability to determine each component in the mixture in the zero-order spectra at *λ*_max_ with maximum accuracy and precision.

The spectrophotometric methods which belong to the fingerprint resolution technique were as follows: the absorptivity centering (a-centering) method [29–31], which was recently published in 2018, and ratio subtraction method (RS) [32–34]. The latter method (RS) was also used with extended ratio subtraction (EXRS) [35, 36], constant multiplication (CM) [37, 38], unified constant subtraction (UCS) [39], and spectrum subtraction (SS) [40, 41] methods to complete the resolution technique.

For best pharmacological action, (CIP) and (FLU) were prepared in their co-formulated otic solution in ratio 12 : 1, respectively. This study aims to apply the six spectrophotometric methods among the fingerprint resolution technique for determination of (CIP) and (FLU) in their challengeable ratio. The recovered zero-order spectra of the cited drugs confirmed their purity. Also statistically, studies were accomplished amongst the presented methods and the official ones, and insignificant differences were remarked. The proposed methods were validated in relation to International Conference on Harmonization (ICH) guidelines [42]. These presented methods could be investigated in quality control laboratories lacking HPLC instruments using the built-in spectrophotometer software without any need for purchasing special software.

## 2. Theoretical Background

### 2.1. Absorptivity Centering (a-Centering) Method

This technique can resolve the mixture of a binary drug (*X* + *Y*) which has shown partially overlapping spectra and interfered at a specific isoabsorptive point *λ*_iso_. It also depends on the choice of two special wavelengths. The first one is *λ*_iso_ and the second one is *λ*_2_ at which the spectrum *Y* is more continuous than the spectrum *X* and the spectrum *X* appears to have no intersection with spectrum *Y* at these *λ*_2_ selected wavelengths.

Before applying this method, some spectral calculation and regression equations should be done, as follows:

Absorptivity inverse (1/*a*_*λ*iso_) at *λ*_iso_ is calculated by applying one of the following software:

(1) Microsoft Excel software:
    Absorbance at *λ*_iso_ for different concentrations of pure *X* or *Y* was measured, and then, each absorbance is divided by its concentration to obtain its correlated absorptivity (*a*_*λ*iso_); consequently, the means of the obtained values and their inverse (1/*a*_*λ*iso_) are calculated as follows:

(1) $$\begin{array}{c} a_{\lambda \text{iso}}=\frac{A_{\left(\lambda \text{iso}\right)}}{\text{conc.}}. \end{array}$$

(2) Spectrophotometer software::
  The ratio spectra of pure *Y* are obtained by dividing different concentrations of *Y* spectra by the *Ý* normalized spectrum of *Y*, and then, a response correlation value (RCV) is calculated at *λ*_iso_ which is a constant value representing [P_*Y*_(*λ*iso)__/A_*Y*_(*λ*iso)__] and has the same value of the absorptivity inverse at *λ*_iso_ verified as follows:

(2) $$\begin{array}{c} \left(\text{RCV}\right)=\frac{P_{Y_{\left(\lambda \text{iso}\right)}}}{A_{Y_{\left(\lambda \text{iso}\right)}}},\\ \;P_{Y_{\left(\lambda \text{iso}\right)}}=\frac{C_{Y}}{C_{Y}}, \end{array}$$

(3) $$\begin{array}{c} P_{Y_{\left(\lambda \text{iso}\right)}}=C_{Y}, \end{array}$$

(4) $$\begin{array}{c} A_{Y_{\left(\lambda \text{iso}\right)}}=a_{\lambda \text{iso}}C_{Y}. \end{array}$$

(5) $$\begin{array}{c} \left(\text{RCV}\right)=\frac{P_{Y_{\left(\lambda \text{iso}\right)}}}{A_{Y_{\left(\lambda \text{iso}\right)}}}\\ \;=\frac{C_{Y}}{a_{\lambda \text{iso}}C_{Y}}\\ \;=\frac{1}{a_{\lambda \text{iso}}}. \end{array}$$

However, *CÝ* is equal to 1 *μ*g/mL; therefore,
Dividing (3) by (4) to gain RCV at *λ*_iso_ gives the following:

#### 2.1.1. Absorptivity Factor

The *D* ^0^ absorption spectra of *X* interfere with the *D* ^0^ absorption spectra of *Y* at *λ*_iso_, and *Y* has a continuing region where the component *X* has negligible absorption at *λ*_2_.

The absorptivity factor [*a*_*λ*iso_/*a*_*λ*2_] between *λ*_iso_ and *λ*_2_ wavelengths is obtained for various concentrations of *Y*, and later, the mean of them is calculated and represented by the absorptivity factor.

#### 2.1.2. Factorized Spectrum (FS′)

Lotfy and Omran [29] recently defined the factorized spectrum of *Y* as “a special spectrum that has absorbance value equal to one at *λ*_iso_ which is acquired by dividing the *D* ^0^ spectra of *Y* by its correlated absorbance value estimated at *λ*_iso_.” Also, the factorized spectrum is applied for the analysis of other mixtures [30, 31]:

(6) $$\begin{array}{c} \left(\text{F}{\text{S}}^{\prime }\right)=\frac{A_{Y}}{A_{Y_{\left(\lambda \text{iso}\right)}}},\\ \left(\text{F}{\text{S}}^{\prime }\right)=\frac{a_{Y}C_{Y}}{a_{Y_{\left(\lambda \text{iso}\right)}}C_{Y}},\\ \left(\text{F}{\text{S}}^{\prime }\right)=\frac{a_{Y}}{a_{Y_{\left(\lambda \text{iso}\right)}}}. \end{array}$$

Spectra of the *X* and *Y* in their linearity range were scanned, and two calibration graphs were constructed between the absorbance of *D* ^0^ spectra and their corresponding concentrations.

Depending on absorptivity inverse value theory and after calculating both the constant and regression parameters, the a-centering method can be applied to recover the *D* ^0^ spectra of each *X* and *Y* in the binary mixture by applying three procedures: first, absorbance calculation at the isoabsorptive point (*A*_*λ*iso_ calculation), followed by recovering the spectrum, and finally, utilizing spectrum subtraction technique.

*(1) Procedure 1 A_λiso_ calculation*. For the more extended spectrum of *Y* in the binary mixture (*X* + *Y*), the absorptivity factor of *Y* was used in order to gain the absorbance of *Y* in the mixture at *λ*_iso_ according to the following equation:

(7) $$\begin{array}{c} \frac{A_{Y\left(\lambda \text{iso}\right)}}{A_{\lambda 2}}=\;\frac{a_{\left(\lambda \text{iso}\right)}C_{Y}}{a_{\lambda 2}C_{Y}},\\ \frac{A_{Y\left(\lambda \text{iso}\right)}}{A_{\lambda 2\left(\text{mix}\right)}}=\;\frac{a_{\left(\lambda \text{iso}\right)}}{a_{\lambda 2}}, \end{array}$$

(8) $$\begin{array}{c} AY_{\left(\lambda \text{iso}\right)}=\left[\frac{a_{\left(\lambda \text{iso}\right)}}{a_{\lambda 2}}\right]\;*\;A_{\lambda 2\left(\text{mix}\right)}, \end{array}$$

where *AY*_(*λ*iso)_ represents the absorbance of *Y* in the mixture at *λ*_iso_; [*a*_(*λ*iso)_/*a*_*λ*2_] represents the absorptivity factor; and *A*_*λ*2(mix)_ represents the absorbance of the mixture at *λ*2.

*(2) Procedure 2 Recovering the spectrum*. The *D* ^0^ spectrum of (*Y*) can be recovered from the binary mixture (*X* + *Y*) by using the normalized or factorized spectrum according to the analyzer's choice. If the analyzer chooses using normalized spectrum, the absorptivity inverse will be multiplied first by the obtained *AY*_(*λ*iso)_ of *Y* and, second, by the normalized spectrum (*Ý*).

However, if the analyzer chooses using the factorized spectrum, the obtained *AY*_(*λ*iso)_ will be multiplied by the factorized spectrum.

*(3) Procedure 3 Utilizing spectrum subtraction technique*. The spectrum subtraction technique [43, 44] was used to recover the *D* ^0^ spectrum of *X*.

### 2.2. Ratio Subtraction (RS) Method

This method is considered as the original method for fingerprint resolution technique through which the *D* ^0^ spectra for the less continuous component *X* in the binary mixture (*X* + *Y*) can be obtained, through the following steps.

First, the mixture (*X* + *Y*) is divided by a certain divisor *Ý* (optimization study will be applied to choose the best divisor) to gain a new curve representing *x*/*Ý* + constant.

Second, the constant is deleted by subtraction *x*/*Ý* + constant − constant.

Third, the *D* ^0^ spectrum (*X*) is acquired by multiplying *Ý* with the previous curve (*x*/*Ý*) *∗* *Ý* = *X*.

### 2.3. Extended Ratio Subtraction (EXRS) Method

This method is considered as extension of the (RS) method through which (EXRS) can obtain the *D* ^0^ spectrum of *Y*, the more continuous spectrum in the binary mixture (*X* + *Y*).

First, the previously obtained *D* ^0^ of *X* within the (RS) method is divided by the certain divisor (*X*′) (optimization study will be applied to choose the best divisor) to get the constant *X*/*X′*.

Second, the mixture (*X* + *Y*) is divided by the same divisor (*X*′) to gain a new curve representing  (*Y*/*X*′)+constant.

Third, the constant is deleted by subtraction *Y*/*X*′+constant − constant.

Fourth, the *D* ^0^ spectrum (*Y*) is acquired by multiplying *X*′ with the previous curve (*Y*/*X*′) *∗* *X*′=*Y*.

### 2.4. Constant Multiplication (CM) Method

This method relates to the (RS) method; it depends on manipulating the obtained constant by applying the (RS) method. This constant *Y*/*Ý* is multiplied by *Ý* to obtain the *D* ^0^ spectrum of *Y*.

### 2.5. Unified Constant Subtraction (UCS) Method

In 2018, Lotfy and Saleh introduced UCS as a complementary method for the (RS) method through which the *D* ^0^ spectrum of *Y* can be obtained after applying the (RS) method using the following steps.

First, the obtained spectrum of *X* is divided by itself to gain constant value one: (*X*/ *X*)=1.

Second, the mixture (*X* + *Y*) is divided by *X* to get a new curve: (*X*+*Y*)/*X*=1+(*Y*/*X*).

Third, the value of one from the previous curve is deleted, and then, it is multiplied with *X*. As a result, the *D* ^0^ spectrum of *Y* is obtained.

### 2.6. Spectrum Subtraction (SS) Method

The *D* ^0^ spectrum of *Y* can be gained after applying the (RS) method by subtracting the obtained *D* ^0^ spectra of *X* from the spectra of the mixture.

## 3. Experimental

### 3.1. Apparatus and Software

Spectrophotometric measurements were carried out on JASCO V-650 double-beam spectrophotometer, using matched 1.00 cm quartz cells. Scans were carried out in the range from 200.0 to 400.0 nm. Spectra were automatically obtained by the JASCO software.

### 3.2. Materials and Reagents

Ciprofloxacin hydrochloride (CIP) was obtained from Unipharma Pharmaceutical Company, Damascus, Syria, and its purity percentage was observed to be 99.95 ± 0.40 according to the BP criteria [6]. Fluocinolone acetonide (FLU) was obtained from Medico Pharmaceutical Company, Homs, Syria, and its purity percentage was observed to be 99.97 ± 0.77 according to the BP criteria [6].

Otovel® otic vials, each vial comprising 0.25 mL solution of ciprofloxacin hydrochloride, amount to 0.75 mg ciprofloxacin and 0.0625 mg fluocinolone acetonide, and they are germ-free, additive-free, pure otic solutions.

Otovel® otic vials were factory-made by Arbor Pharmaceuticals under license of Laboratorios SALVAT in Barcelona (Spain).

Methanol of analytical grade was purchased from Panreac, Barcelona, Spain.

### 3.3. Solution Preparation

Standard stock solutions containing 1000.0 *μ*g/mL of (CIP) and (FLU) were made separately in methanol.

(CIP) and (FLU) working solutions (each, 50.0 *μ*g/mL) were prepared by diluting the previous stock solutions with methanol.

### 3.4. Spectral Characteristics

For studying the spectral characteristic of (CIP), (FLU), and the binary mixture of (CIP) + (FLU), the absorption spectra were scanned against methanol as blank over the range of 200, 0–400, 0 nm for the 10.0 *μ*g/mL of (CIP), 10.0 *μ*g/mL of (FLU), and laboratory mixture (CIP + FLU) comprising 5.0 *μ*g/mL of each in methanol.

## 4. Procedure

### 4.1. Linearity and Construction of Calibration Curves

The *D* ^0^ absorption spectra were recorded for the standard solutions of both (CIP) and (FLU) equivalent to 3.0–15.0 *μ*g/mL, respectively, prepared separately in the solvent mixture. Calibration curves were constructed for both (CIP) and (FLU) by plotting the absorbance of *D*_max_^0^ (CIP at 278.0 nm and (FLU) at 238.0 nm) against the concentrations in *μ*g/mL of (CIP) and (FLU), separately computed and used for all methods.

Laboratory-prepared working solutions of (FLU) in concentration <3.0 *μ*g/mL need to use the spectrum addition as the sample enrichment technique as follows: spectrum of pure (FLU) (4.0 *μ*g/mL) was added to that of those mixtures in order to achieve the linearity of (FLU), to avoid the deviation of Beer's law which occurs in the case of low absorptivity for the minor component presented in the mixtures.

#### 4.1.1. a-Centering Method of Both (CIP) and (FLU)

Some spectral calculations were computed as follows:

- Absorptivity factor of pure (CIP) (*A*_248.2nm_/*A*_318.2nm_) for different concentrations of (CIP) was calculated at 318.2 nm; the absorbance value for (FLU) at 318.2 nm is zero.
- The stored absorption spectra of (CIP) were divided by NS_CIP_′, the normalized spectrum, to obtain ratio spectra of (CIP); then, the amplitudes at 248.2 nm (*λ*_iso_) (*P*_CIP(248.2nm)_) were recorded.
- The response correlation value (RCV) was calculated for different concentrations of pure (CIP) as follows:

(9) $$\begin{array}{c} \text{RCV}=\frac{P_{\text{CIP}\left(248.2\text{ nm}\right)}}{A_{\text{CIP}\left(248.2\text{ nm}\right)}}. \end{array}$$

(iv) Factorized spectrum of FS_CIP_′ is acquired by dividing the absorption spectrum of (CIP) by its absorbance value estimated at 248.2 nm.

#### 4.1.2. (RS) Method of (FLU)

Special optimization study for choosing the best divisor of (CIP)′ was done. Spectra of pure (FLU) were divided by the best spectrum divisor of (CIP)′, and the constant obtained from the plateau region is subtracted. The *D* ^0^ spectrum of (FLU) is acquired by multiplying (CIP)′ with the previous curve.

#### 4.1.3. (EXRS) Method of (CIP)

Special optimization study for choosing the best divisor of (FLU)′ was done. Spectra of pure (CIP) were divided by the best spectrum divisor of (FLU)′, and the constant obtained from the plateau region is subtracted. The *D* ^0^ spectrum of (CIP) is acquired by multiplying FLU′ with the previous curve.

#### 4.1.4. CS Method of (CIP)

Spectra of pure (CIP) were divided by the best spectrum divisor of (CIP)′, the constant was obtained (CIP/CIP′) and multiplied by the divisor of (CIP′), and the *D* ^0^ spectrum of (CIP) is acquired.

#### 4.1.5. UCS and SS Methods of (CIP)

The calibration curve is constructed relating the absorbance of zero-order spectra of (CIP) at 278.0 nm, and the corresponding concentrations and regression equations are computed.

### 4.2. Application to Laboratory-Prepared Mixtures

Different aliquots equivalent to 3.0–15.0 *μ*g/mL for both (CIP) and (FLU) were separately prepared as two separate series of 10 mL volumetric flasks via appropriate dilution of their respective working solutions (50.0 *μ*g/mL of both (CIP) and (FLU)) using methanol. The prepared solutions were scanned in the range of 200.0–400.0 nm against methanol as a blank and stored in the computer.

For mixtures containing (FLU) in concentration less than 3.0 *μ*g/mL, standard spectrum addition is used via added spectrum of 4.0 *μ*g/mL of pure standard (FLU) to every recorded spectrum of those mixtures by using JASCO software.

By measuring the difference between the total and added concentrations of (FLU), the claimed concentration of (FLU) in every mixture was acquired.

Mixtures containing (FLU) in concentration equals or more than 3.0 *μ*g/mL are not required for spectrum adding technique.

The procedure previously mentioned for every technique was followed to obtain the concentrations of (CIP) and (FLU) within the laboratory-prepared mixtures.

### 4.3. Application to Pharmaceutical Formulation

The contents of one vial are dripped precisely into 100 mL volumetric flasks and then completed to the mark with methanol, and a solution with final concentration claiming 75.0 *μ*g/mL of (CIP) and 6.25 *μ*g/mL of (FLU) was obtained. The previous solution was filtered through a Whatman filter paper, and the volume was completed to 100 mL with methanol. To obtain a solution with final concentration claiming 12.0 *μ*g/mL of (CIP) and 1.0 *μ*g/mL of (FLU), a proper dilution was done.

The final concentration of (FLU) is 1.0 *μ*g/mL, so the recorded spectrum of pure (FLU) (4.0 0 *μ*g/mL) was summed to those of the working solutions to achieve the linearity range of (FLU). The procedure described previously under each method was done to calculate the concentration of (CIP) and FLU in Otovel®.

## 5. Results and Discussion

Analytical methods for the determination of binary mixture without previous separation are of interest to quality control (QC) laboratories and national regulatory authorities (NRA) around the world.

**Figure 2:**
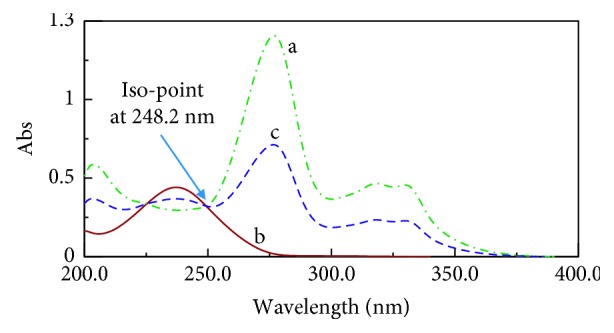
Zero-order spectra of (a) (CIP) (10.0 *μ*g/mL), (b) FLU (10.0 *μ*g/mL), and (c) a mixture containing 5.0 *μ*g/ml of (CIP) and FLU showing the isoabsorptive point.

The absorption spectra of (CIP) and (FLU) show partial overlap with the isoabsorptive point at 248.2 nm (Figure 2).

**Figure 3:**
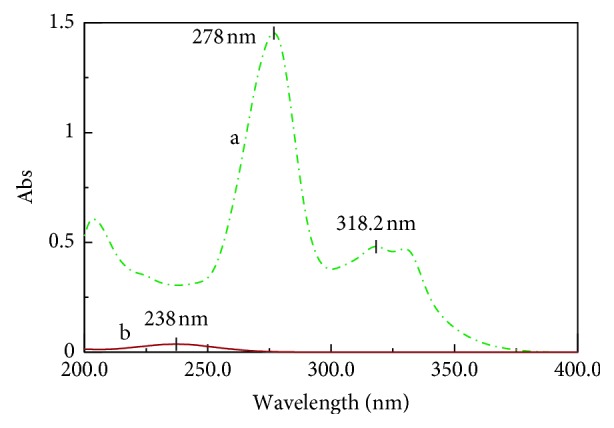
Zero-order spectra of (a) (CIP) (12.0 *μ*g/mL) and (b) FLU (1.0 *μ*g/mL) showing the ratio of the dosage form.

The application of the fingerprint resolution method for the determination of (CIP) and (FLU) in their binary mixture was investigated in this study within this method, and the *D* ^0^ spectra of the cited drugs were obtained by applying six spectrophotometric approaches regarding the synchronous evaluation of (CIP) and (FLU) in otic solution and in their pure form with a challengeable ratio of 12 : 1, respectively. Thus, (CIP) acts as the major component, while the (FLU) is the minor ones. The *D* ^0^ spectra of (CIP) and (FLU) presented intervention with partial overlap, and also, their spectra display isoabsorptive point, as shown in (Figure 3). Regrettably, (CIP), which is the main existing analyte within the Otovel® otic solution, is also of upper absorptivity, which complicates the (FLU) minor component estimation at its *λ*_max_, whereas (FLU) displays only slight absorbance specifically once appearing in minor concentrations. To solve this problem, spectrum addition technique was successfully applied.

Therefore, different spectral manipulating techniques have been applied for analysis of the cited drugs in their laboratory mixtures consisting of different proportions of the cited drugs and their combined otic formulation. The a-centering method was successfully applied to gain the concentration of both drugs, while the (RS) method could obtain the concentration of less extended ones (like FLU) only. Therefore, four complementary methods (EXRS), (CM, UCS, and SS) connected with the (RS) method to complete the resolution of this binary mixture. As a result, the concentration of (CIP) was obtained.

An optimization study was done to obtain the best divisor of (CIP) and (FLU) for (RS) and (EXRS) methods, respectively, as shown in Table 1.

This study aimed mainly to develop procedures with satisfactory precision and accuracy for determining the binary mixture components presented in Otovel®.

### 5.1. Absorptivity Centering (a-Centering) Method

Studying the spectral characteristic of (CIP), (FLU), and a binary mixture of them (CIP + FLU) has shown partially overlapping spectra and interference at a specific isoabsorptive point *λ*_iso_. In addition, (CIP) spectra are more continuous than (FLU) and showed wavelength at 318.2 nm, which does not contribute with (FLU), as shown in Figure 3.

The a-Centering method can be applied to recover the zero-order spectra of both (CIP) and (FLU) in the binary mixture (CIP + FLU) by applying three procedures: first, *A*_*λ*iso_ calculation is done, followed by recovering the spectrum of (CIP), and finally, utilizing the spectrum subtraction technique for recovering *D* ^0^ spectra of (FLU).

Before applying this method, some spectral calculation and regression equations were acquired as follows:

- Calculating absorptivity inverse value (1/*a*_*λ*iso_) by using Excel software: absorbance at 248.2 nm (*λ*_iso_) was measured using the *D* ^0^ spectra of (CIP) ranged between 3.0–15.0 *μ*g/mL to get its corresponding absorptivity; it was found from equation (1) that *a*_CIP(248.2nm)_ equals 0.027, and as a result, absorptivity inverse (1/*a*_CIP(248.2nm)_) was 36.98. Getting absorptivity inverse value (1/*aλ*_iso_) by using spectrophotometric software: the scanned *D* ^0^ spectra of (CIP) (1.0–15.0 *μ*g/mL) were divided by the normalized spectrum of (CIP)′, and amplitudes of the obtained ratio spectra of (CIP) at *λ*_iso_(*P*_CIP(248.2nm)_) were registered. Using equation (5), RCV between *P*_CIP(248.2nm)_ of various concentrations of (CIP) versus its corresponding *A*_CIP(248.2nm)_ registered the value 36.98 and represented the same value of absorptivity inverse (1/*aλ*_iso_).
- Absorptivity factor (*a*_248.2nm_/*a*_318.2nm_) was calculated and registered 0.68.
- The normalized and factorized spectrum of (CIP) were obtained.
- (FLU) determination by the spectrum addition technique: the *D* ^0^ spectrum 4.0 *μ*g/mL of pure standard (FLU) using UV spectrum software was added to those of all laboratory-prepared mixtures containing (FLU) in concentration less than 3.0 *μ*g/mL and in pharmaceutical preparation. By measuring the difference between the total and added concentrations of (FLU) in each mixture, the real concentration of (FLU) was acquired.
- Spectra of the (CIP) and (FLU) in their linearity range were scanned, and two calibration graphs were built up between the *D* ^0^ spectrum of (CIP) at 278.0 nm and that of (FLU) at 238.0 nm against their corresponding concentrations.

**Figure 4:**
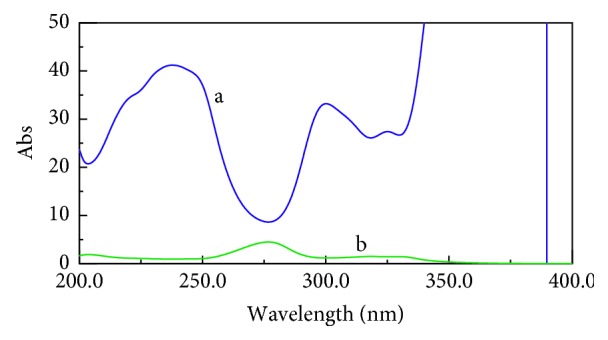
5.0 *μ*g/ml of (CIP) spectra representing. (a) Absorptivity inverse (1/a) of (CIP), and (b) Factorized spectrum of (CIP) (FS′).

After calculating this constant parameter values and regression parameters, we can apply the a-centering method in three steps:

 
*Step 1*. Obsorbance calculation at the isoabsorptive point [*A*248.2nm]. Laboratory mixtures were measured at 318.2 nm, then absorbance of (CIP) at *λ*_iso_ 248.2 nm was gained in these mixtures through the following equation:

(10) $$\begin{array}{c} A_{248.2\text{nm}}=\;\left(\frac{a_{248.2\text{nm}}}{a_{318.2\text{nm}}}\right)\times A_{318.2\text{nm}\left(\text{mix}\right)}, \end{array}$$

  where *A*_248.2nm_ represents the absorbance of (CIP) in the mixture at *λ*_iso_ 248.2 nm and (*a*_248.2nm_/*a*_318.2nm_) represents the absorptivity factor, which is equal to 0.681.
 
*Step 2*. Spectrum recovery. In order to recover the *D* ^0^ spectrum of (CIP) in every mixture, multiply the gained  *A*_248.2nm_ of (CIP) in every laboratory mixture by absorptivity inverse [1/*a*_CIP(248.2nm)_] calculated at *λ*_iso_ 248.2 nm, which was found to be 36.98, and then the result is multiplied by the (CIP)′ normalized spectrum, as shown in Figure 4.

(11) $$\begin{array}{c} D_{\text{recovered}\left(\text{CIP}\right)}^{0}=A_{248.2\text{nm}\left(\lambda \text{iso}\right)}\times \left[\frac{1}{a_{\text{CIP }\left(248.2\text{nm}\right)}}\right],\\ D_{\text{recovered}\left(\text{CIP}\right)}^{0}=A_{248.2\text{nm}\left(\lambda \text{iso}\right)}\times 36.98, \end{array}$$

  or by directly multiplying the obtained *A*_248.2nm(*λ*iso)_ by the FS_CIP_′ factorized spectrum of (CIP) to get the *D* ^0^ absorption spectrum of (CIP) in each mixture, as shown in Figure 4:

(12) $$\begin{array}{c} D_{\text{recovered}\left(\text{CIP}\right)}^{0}=A_{248.2\text{nm}\left(\lambda \text{iso}\right)}\times {\text{FS}}_{\text{CIP}}^{\prime }. \end{array}$$

 
*Step 3*. Spectrum subtraction. To gain the *D* ^0^ spectrum of (FLU) in the mixture, subtract the gained *D* ^0^ spectrum of (CIP) from the *D* ^0^ absorption spectrum of the corresponding mixture.

From the above, (CIP) and (FLU) concentrations were estimated using their regression equations at their *λ*_max_.

The absorptivity centering method (a-Centering) via the normalized spectrum involves several manipulating steps, so it is more convenient when applied via the factorized spectrum.

This approach has an improvement that it is able to determine the concentrations of both mentioned drugs by their unified regression equation, and the use of normalized and factorized spectra minimizes the mistake in measurement of concentrations of the minor component (FLU) in binary mixtures.

### 5.2. Ratio Subtraction (RS) Method

In the ratio subtraction method, the important step is to choose the best concentration's divisor. First, an optimization study was done by taking three concentrations of (CIP) within their linearity (4.0, 8.0, and 12.0 *μ*g/mL), then the laboratory-prepared mixtures were divided separately by these three concentrations, the constant (CIP/CIP′) was measured in the plateau region (300.0–330.0 nm), and the results obtained were compared to the theoretical results and judged according to the minimum average absolute difference between theoretical and acquired constants. The concentration 12.0 *μ*g/mL of (CIP) gave the best result, as shown in Table 1.

(FLU) only was estimated by this technique through dividing the spectra of the laboratory-prepared mixtures (CIP + FLU) by 12.0 *μ*g/mL (CIP′) divisor in order to gain a new curve representing (CIP/CIP′)+(FLU/CIP′)=(FLU/CIP′)+constant.

**Figure 5:**
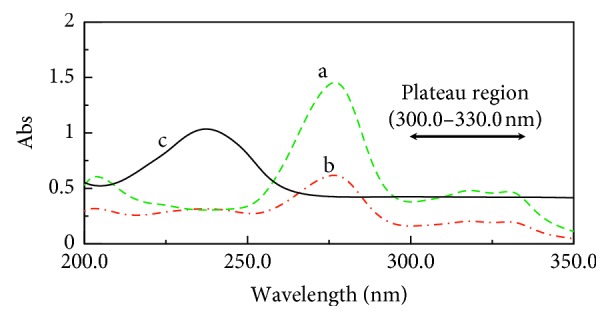
(a) Zero-order absorption spectrum of (CIP)′ (12.0 *μ*g/mL) as a divisor; (b) zero-order absorption spectrum of a mixture of (CIP) and FLU (5.0 *μ*g/ml each); (c) ratio spectrum of this mixture using of (CIP)′ (12.0 *μ*g/mL) as a divisor showing in the plateau region (300.0–330.0 nm).

The constant mentioned above was determined in the plateau region (300.0–330.0 nm) and subtracted from the previous equation, as shown in Figure 5. Finally, by multiplying FLU/CIP′ with divisor 12.0 *μ*g/mL (CIP′), the *D* ^0^ spectra of (FLU) will be obtained from the binary mixture (CIP + FLU).

For the (FLU) determination in the laboratory-prepared mixtures containing (FLU) in concentration less than 3.0 *μ*g/mL and in pharmaceutical preparations, the spectrum addition technique was applied as mentioned above in a-centering method.

The (RS) method is applied through four manipulating steps for determining the less extended component (FLU) at its *λ*_max_, and thus supplementary methods are required to complete the determination of the other component (CIP) of the binary mixture. In addition, selecting the best divisor and plateau region and using the pure form of the more extended component as a divisor are required in order to complete the analysis; however, the (RS) method can applied without need for special software or derivative steps.

In order to complete the resolution process after applying the (RS) method and acquire the *D* ^0^ spectra of (FLU) from the binary mixtures (CIP) and FLU, one of the following methods was combined with the (RS) method and successfully applied for the determination of (CIP) and to extract its zero-order absorption spectrum, thereby getting its concentrations using regression equation at its maxima:

#### 5.2.1. Extended Ratio Subtraction (EXRS) Method

The D^0^ spectra of (FLU) obtained by the (RS) method was divided by a divisor (FLU′) 12.0 *μ*g/mL, which was found to be the best divisor obtained by optimization of three concentrations of (FLU) (4.0, 8.0, and 12.0 *μ*g/mL) within their linearity.

The constant value of FLU/FLU′ was estimated at the 220.0–260.0 nm plateau region.

**Figure 6:**
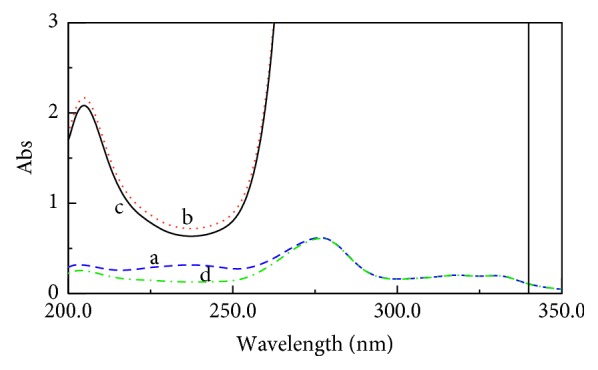
The spectra obtained by using the EXRS method. (a) A mixture containing 5.0 *μ*g/ml of each (CIP) and FLU; (b) the mixture after divided by 12.0 *μ*g/mL of FLU′; (c) the mixture after subtracting the constant; (d) the pure (CIP) obtained after multiplied by FLU′.

The spectra of laboratory-prepared mixtures were divided by a divisor (FLU′) 12.0 *μ*g/mL, and the ratio spectra were gained, the constant FLU′ was subtracted from the previous ratio spectra, and later those ratio spectra were multiplied by FLU′ in order to obtain *D* ^0^ of (CIP), as shown in Figure 6.

The (EXRS) method consists of four manipulating steps: choose the best divisor, get the ratio spectra, subtract the constant, and multiply by the divisor.

These several steps make the (EXRS) method more time-consuming while applying for binary mixture analysis.

The advantages of using the (EXRS) method in analysis of the binary mixture are that no special software is required for applying this technique, accurate results are obtained through it, and also it recovers the pure *D* ^0^ spectra of the (CIP) component with no interference.

The disadvantages of this method are that accurate measurement of a constant is required and using the pure form of the divisor and choosing the best concentration of the divisor.

#### 5.2.2. Constant Multiplication (CM) Method

The constant CIP/CIP′ obtained by the (RS) method was multiplied by (CIP)′ to gain the *D* ^0^ spectra of (CIP).

This complementary method (CM) does not require using a divisor to complete the analysis of binary mixture, and also just two steps are required to complete the determination of (CIP), which make it a simple method when applied. But, the only limitation is that the calculation of (CIP) is dependent on the recorded constant CIP/CIP′, so the risk of error results is increased.

#### 5.2.3. Unified Constant Subtraction (UCS) Method

The spectra of laboratory-prepared mixtures (CIP + FLU) was divided by an (FLU)′ divisor, obtained from the (RS) method, in order to obtain a new curve representing (CIP/FLU′)+(FLU/FLU′)=(CIP/FLU′)+constant. The constant value is one in this equation; if the value one is subtracted from the previous equation and then multiplied by (FLU)′, the *D* ^0^ spectra of (CIP) are obtained. This complementary method (UCS) does not require an optimization study to choose the best divisor to obtain the *D* ^0^ of (CIP), and also it does not require any pure form of (CIP) to complete the determination of the binary mixture components, but the only limitation of this method is more time consumption since it consists of three steps.

#### 5.2.4. Spectrum Subtraction (SS) Method

The spectra of (CIP) in zero-order form were obtained from the binary mixture (CIP + FLU) by subtracting the spectra of (FLU), which was obtained by the (RS) method from the spectra of the binary mixture. This approach has advantage of being simple and having a few steps to get the *D* ^0^ spectra of (CIP) without requirement of a special divisor or a recorded constant, but the limitation of this method is the noise interfering when acquired (CIP) by subtraction.

Calibration equations and concentration ranges for all the proposed methods are mentioned in Table 2. The proposed techniques were successfully effectively applied for (CIP) and (FLU) analysis in their laboratory-prepared mixtures containing different ratios of (CIP) and (FLU), as shown in Table 3. The proposed procedures were also applied for the determination of Otovel® ear drops after applying spectrum adding techniques, as shown in Table 3.

## 6. Method Validation

The six proposed approaches were validated in compliance with the ICH guideline [40] with respect to methods' range, linearity, accuracy, and precision, as shown in Table 2. The suggested methods (a-centering, (EXRS), CM, UCS, and SS) have the same results for determination of (FLU), and a-centering and (RS) methods have the same results for determination of (CIP), and this is because all methods regained the zero-order absorption spectra of the studied drugs. Thus, the concentration of each drug was calculated using calibration curves representing absorbance at its maxima versus its corresponding concentration. Table 3 shows the results obtained from the analysis of laboratory-prepared mixtures containing different ratios of (CIP) and (FLU), ensuring the selectivity of the proposed methods where satisfactory results were obtained over the calibration range. The proposed methods were also performed for the determination of the drugs in Otovel® otic solution, where satisfactory results were obtained, as shown in Table 3.

## 7. Statistical Analysis

Table 4 shows the calculated *t* and *F* values for the statistical comparison of the results obtained by the proposed methods and the official ones [6].  Table 5 shows one-way ANOVA statistical comparison of the results obtained by the proposed approaches and the official methods when applied to Otovel® otic solution. The results from both tables showed that there was no significant difference between the proposed approaches and the official ones with respect to accuracy and precision.

### 7.1. Comparative Study between the Developed and Reported Spectrophotometric Methods [28]

Table 6 illustrates the advantages and limitations of each of the developed fingerprint spectrophotometric methods, and it was clear that all the developed methods have several unified advantages that they are able to recover and determine the component of both at their *λ*_max_, so maximum accuracy, precision, and reproducibility without any need for purchasing special software. In addition, the recovered spectra were typical to that of pure components which confirm the spectral profile of each component of interest. These advantages besides the minimum limitation made them superior over the reported spectrophotometric methods [28].

## 8. Conclusion

This report delivered the utility of new, uncomplicated, green, economic spectrophotometric approaches for the determination of the recently delivered binary combination of (CIP) and (FLU). The a-centering method have the optimum efficiency power to determine both analytes depending on the isosbestic point theory; however the (RS) method could determine only one analyte, but supplementary methods (EXRS), (CM, UCS, and SS) connected with (RS) solved this problem; therefore, the two analytes could be determined with accurate results. The supplementary methods differed in the number of handling steps where (EXRS) involves four handling steps, CM consists of two steps, UCS consists of three steps, and SS consists of only one step and both methods were applied using one divisor only. Furthermore, while operating with these techniques, which belong to the fingerprint resolution technique, all determinations were done after recovering the zero-order spectra for each component from their mixture that allow estimation of each component in their zero-order regression equation. As a final point, it is seen that the suggested fingerprint resolution technique is the only spectrophotometric method which could be used in resolving complex matrices and testing the purity of the resolved spectra. These proposed methods could be utilized within the routine analysis in QC research laboratory.

## Figures and Tables

**Table 1:** Selection of the best divisor of (CIP) and FLU by the optimization study.

Mix no.	(CIP) divisors					
	4.0 *μ*g/mL		8.0 *μ*g/mL		12.0 *μ*g/mL	
	Post^*∗*^	Rec^*∗∗*^	Post^*∗*^	Rec^*∗∗*^	Post^*∗*^	Rec^*∗∗*^

1	1.250	1.277	0.625	0.632	0.417	0.422
2	2.500	2.545	1.250	1.259	0.833	0.842
3	1.000	1.020	0.500	0.537	0.333	0.326
4	1.000	1.028	0.500	0.503	0.333	0.331
5	2.000	2.044	1.000	0.980	0.667	0.645

AAD^*∗∗∗*^	0.033		0.015		0.009	

Mix no.	FLU divisors					
	4.0 *μ*g/mL		8.0 *μ*g/mL		12.0 *μ*g/mL	
	Post^*∗*^	Rec^*∗∗*^	Post^*∗*^	Rec^*∗∗*^	Post^*∗*^	Rec^*∗∗*^

1	1.250	1.352	0.625	0.635	0.417	0.421
2	1.000	1.041	0.500	0.488	0.333	0.343
3	2.500	2.452	1.250	1.271	0.833	0.845
4	2.000	2.013	1.000	1.002	0.667	0.668
5	1.000	1.018	0.500	0.482	0.333	0.331
AAD^*∗∗∗*^	0.044		0.013		0.006	

The values represent the constant at the plateau regions (300.0–330.0 nm) for (CIP) and (220.0–260.0 nm) for FLU. ^*∗*^Post: postulated value of constant; ^*∗∗*^Rec: recorded value of constant; ^*∗∗∗*^AAD: average absolute difference between postulated and recorded constant values.

**Table 2:** Assay parameters and validation sheets for pure cited drugs at their maxima.

Parameter	CIP^*∗*^	FLU^*∗∗*^
Wavelength (nm)	278.0	238.0
*N*	7	7
Range (*μ*g/mL)	3.0–15.0	3.0–15.0
Intercept	−0.0007	−0.0011
Slope	0.1209	0.0357
Correlation coefficient	0.9999	0.9999
Accuracy^a,b^	100.08 ± 0.51	99.79 ± 0.54
Repeatability^a,c^	0.43	0.82
Interday precision^a,c^	1.19	1.03

^*∗*^a-Centering, EXRS, CM, UCS, and SS. ^*∗∗*^a-Centering and RS. ^a^Average of three experiments. ^b^Mean ± standard deviation. ^c^RSD% of concentrations (4.0, 8.0, and 12.0 *μ*g/mL of both CIP and FLU).

**Table 3:** Analysis of laboratory-prepared mixtures and the dosage form by the proposed spectrophotometric methods.

CIP : FLU^a^ (*μ*g/mL)	CIP						FLU		
	Recovery %								
	a-Centering		EXRS	CM	UCS	SS	a-Centering		RS
	NS^*∗*^	FS^*∗∗*^					NS^*∗*^	FS^*∗∗*^	
5 : 5	98.00	99.80	101.78	99.80	102.04	100.60	97.80	99.80	100.06
10 : 4	99.90	100.10	99.80	101.09	100.59	99.90	100.05	100.05	100.00
4 : 10	100.05	100.03	100.15	99.75	100.75	98.58	99.90	99.80	100.02
4 : 8	100.15	101.78	100.08	100.08	99.75	101.28	99.84	100.01	98.63
8 : 4	99.88	100.04	99.88	98.75	101.25	100.38	99.75	99.50	100.05
12 : 5^b^	100.03	99.99	100.04	100.05	100.03	100.00	100.06	99.80	99.80
Mean^c^ ± SD	99.67 ± 0.82	100.29 ± 0.74	100.29 ± 0.74	99.92 ± 0.75	100.73 ± 0.83	100.12 ± 0.90	99.57 ± 0.87	99.83 ± 0.20	99.76 ± 0.56
^a,d^Otovel® batch no. 24338-080-14	100.03 ± 0.56	99.81 ± 0.36	99.66 ± 0.54	99.44 ± 0.61	99.61 ± 0.52	99.75 ± 0.78	100.27 ± 1.20	99.45 ± 1.31	99.37 ± 1.21

^a^Average of three experiments. ^b^Ratio present in Otovel® before subtraction of the added (FLU) spectrum (4 *μ*g/mL). ^c^Mean of the percentage recovery of all laboratory-prepared mixtures and standard deviation. ^d^Mean and standard deviation of the percentage recovery of Otovel®. ^*∗*^NS results were obtained by using the normalized spectrum. ^*∗∗*^FS results were obtained by using the factorized spectrum.

**Table 4:** Statistical comparison between the results obtained by the proposed spectrophotometric methods and official methods [6] for the determination of CIP and FLU in Otovel® otic solution.

Methods	CIP							FLU			
	a-Centering		EXRS	CM	UCS	SS	BP^a^	a-Centering		RS	BP^a^
	NS^*∗*^	FS^*∗∗*^						NS^*∗*^	FS^*∗∗*^		
Mean	100.03	99.81	99.66	99.44	99.61	99.89	99.85	100.27	99.45	99.37	99.73
SD	0.56	0.36	0.54	0.61	0.52	0.64	0.32	1.20	1.31	1.21	0.61
Variance	0.31	0.13	0.29	0.37	0.27	0.42	0.10	1.45	1.73	1.46	0.37
*N*	6	6	6	6	6	6	6	6	6	6	6
*t*-Test^b^	0.86	0.18	0.73	1.46	0.96	0.14	—	0.97	0.48	0.66	—
*F* value^c^	3.01	1.27	2.80	3.55	2.63	4	—	3.87	4.61	3.90	—

^a^The BP method for (CIP) is HPLC, while ^a^the BP method for FLU is the absorption method. ^b^The corresponding tabulated value of Student's *t*-test is equal to 2.23 at *p*=0.05. ^c^The corresponding tabulated value of *F* is equal to 5.05 at *p*=0.05. ^*∗*^NS results were obtained by using the normalized spectrum. ^*∗∗*^FS results were obtained by using the factorized spectrum.

**Table 5:** Results of one-way ANOVA for comparison of the proposed and the official methods [6] for determination of (CIP) and FLU in Otovel® otic solution.

Source of variation		Degree of freedom	Sum of squares	Mean square	*p* value^a^	*F* value^a^	*F* critical^a^
CIP	Between columns	6	1.41	0.24	0.53	0.87	2.37
	Within columns	35	9.49	0.27			
	Total	41	10.90				

FLU	Between columns	3	2.97	0.99	0.51	0.79	3.10
	Within columns	20	25.07	1.25			
	Total	23	28.05				

^a^There was no significance difference between the methods using one-way ANOVA at *p* < 0.05.

**Table 6:** Advantages and limitations of each fingerprint method used for the analysis of co-formulated otic solution of ciprofloxacin and fluocinolone acetonide in their challengeable ratio.

Method	Advantages	Limitations
a-Centering via normalized spectrum	(1) Measurement was done in zero order (2) It is able to recover and determine the component of both at their *λ*_max_, so it gives maximum accuracy and reproducibility (3) Obtaining spectra typical to that of pure components which confirm the spectral profile of each component of interest (4) No need for special software (5) Needs calculation of one factor (6) Easy and accurate	(1) Four manipulation steps (2) Calculation of two factors (3) Presence of isopoints (4) Preparing the normalized spectrum via spectrophotometer software

a-Centering via factorized spectrum	(1) Measurement was done in zero order (2) It is able to recover and determine the component of both at their *λ*_max_, so it gives maximum accuracy and reproducibility (3) Obtaining spectra typical to that of pure components which confirm the spectral profile of each component of interest (4) No need for special software (5) Needs calculation of one factor (6) Simple preparation of the factorized spectrum (7) Easy and accurate	(1) Three manipulation steps (2) Presence of isopoints (3) Preparing the factorized spectrum via spectrophotometer software

Ratio Subtraction (RS)	(1) It is able to determine the nonextended component at its *λ*_max_ with maximum accuracy and reproducibility (2) Obtaining spectra typical to that of pure components which confirm the spectral profile of each component of interest (3) No need for special software	(1) Three manipulation steps (2) Recovery of zero order for the less extended spectra only (3) Availability of the pure drug of the component with extended spectrum as a divisor (4) Optimization study for choosing the best concentration of the divisor

Extended Ratio Subtraction (EXRS)	(1) It is able to determine the extended component at its *λ*_max_ with maximum accuracy and reproducibility (2) Obtaining spectra typical to that of pure components which confirm the spectral profile of each component of interest (3) No need for special software	(1) Four manipulation steps (2) Recovered the *D*_0_ spectra of the more extended one only (3) Accurate measuring of a constant is required (4) Need for the pure drug of the component with the nonextended spectrum as a divisor (5) Optimization study was applied to choose the best concentration of the divisor

Constant Multiplication (CM)	(1) No need for special software (2) No need for isopoint (3) No need for calculated factor	(1) Two manipulation steps (2) Accurate measuring of a constant is required (3) Need for the pure sample of the drug of interest as a divisor (4) Optimization study was applied in order to choose the best concentration of the divisor

Unified Constant Subtraction (UCS)	(1) Measurement was done on ratio spectra (2) No need for isopoint (3) No need for pure form, divisors, or calculated factors	(1) Three manipulation steps (2) Complementary method for the RS method

Spectrum Subtraction (SS)	(1) No need for isopoint (2) No need for pure form, divisors, or calculated factors (3) One-step manipulation	(1) Applied only as a complementary method

## Data Availability

The data used to support the findings of this study are included within the article.
